# Preliminary Study on the Imbalance Between Th17 and Regulatory T Cells in Antiphospholipid Syndrome

**DOI:** 10.3389/fimmu.2022.873644

**Published:** 2022-05-06

**Authors:** Huanhuan Yan, Baochen Li, Rui Su, Chong Gao, Xiaofeng Li, Caihong Wang

**Affiliations:** ^1^Department of Rheumatology, The Second Hospital of Shanxi Medical University, Taiyuan, China; ^2^Pathology, Joint Program in Transfusion Medicine, Brigham and Women’s Hospital/Children’s Hospital, Harvard Medical School, Boston, MA, United States

**Keywords:** primary antiphospholipid syndrome (PAPS), secondary antiphospholipid syndrome (SAPS), T helper 17(Th17) cells, regulatory T(Treg) cells, cytokines

## Abstract

**Objective:**

Patients with antiphospholipid syndrome (APS) have immune cell abnormalities that remain poorly understood. This study compared primary APS (PAPS) and secondary APS (SAPS) patients with healthy controls with respect to peripheral blood lymphocytes, CD4+T cell subsets, and cytokine levels. The correlation between antiphospholipid antibody titres and T helper 17 (Th17) and T regulatory (Treg) cell subsets was also analyzed, together with the correlations between cytokine profiles and the clinical characteristics of APS patients.

**Methods:**

The retrospective study population consisted of 67 APS patients (12 with PAPS, 55 with SAPS) and 40 healthy controls. Absolute numbers of peripheral blood lymphocyte subsets and CD4+ T cell subsets were detected by flow cytometry, and serum cytokine levels by flow cytometry bead array.

**Results:**

Patients with SAPS had lower absolute values of T, B and CD4+T cells than the healthy control group, while only natural killer (NK) cell levels were decreased in patients with PAPS. Absolute numbers of T, B, NK, and CD4+T cells were significantly higher in the PAPS than SAPS group. The trends in CD4+T cell subsets were the same in PAPS and SAPS patients as in healthy controls, with increased Th1, decreased Th2, and decreased Treg levels, and thus an increased Th17/Treg ratio. Th2, Th17, and Treg cell counts were higher in the PAPS than SAPS group. Cytokine analysis showed that only IL-10 levels differed between the two APS groups. However, the levels of all of the studied cytokines were higher in APS patients than healthy controls, and correlated with the clinical characteristics of the patients. In the PAPS group, the titres of two autoantibodies correlated positively with the Th17/Treg ratio and negatively with the levels of D-dimer and Treg subsets.

**Conclusions:**

Our study clearly showed that APS patients have immune disturbances, the most prominent of which is an increase in the Th17/Treg ratio, due to a decrease in the number of Treg cells. These abnormalities may be involved in the occurrence and progression of APS. An additional finding was a higher level of peripheral blood lymphocytes in PAPS than SAPS patients, which may be related to the immunosuppressive treatment of SAPS patients.

## Introduction

Antiphospholipid syndrome (APS) refers to an autoimmune disease characterized by multiple arterial or venous thrombosis, pregnancy complications, and/or thrombocytopenia with positivity for antiphospholipid antibodies (aPL), including lupus anticoagulants (LA), anticardiolipin antibodies (aCL), and anti-β2 glycoprotein-I (aβ2GPI) (1–4). The estimated incidence and prevalence of APS ranged between 1 and 2 cases per 100,000 and 40 and 50 cases per 100,000 respectively (5). Patients may also develop kidney disease, digestive tract ischemia, pulmonary manifestations, and cardiac and neuropsychiatric symptoms (6, 7). The most common complication of obstetric APS (OAPS) is recurrent miscarriage in the first trimester, which has been attributed to inhibition of the proliferation of trophoblastic cells (8). APS is classified into primary antiphospholipid syndrome (PAPS) and secondary antiphospholipid syndrome (SAPS) based on the presence or absence of other autoimmune diseases, mainly systemic lupus erythematosus (SLE), Sjögren’s syndrome (SS) and rheumatoid arthritis (RA) (1, 6). The concomitant presence of other autoimmune diseases complicates disease management. In addition, PAPS has several features not seen in SAPS, including low serum C3 and C4 levels, but elevated C3a-desArg and C4a levels, suggesting that complement activation is involved in the pathogenesis of PAPS (9). Other immunological similarities and differences between PAPS and SAPS, including those involving peripheral blood lymphocyte subsets, have not been investigated.

Several factors are involved in the development of APS, among which the inflammatory response is crucial. In APS, aPL activates Toll-like receptors (TLR) and, in turn, the innate immune pathway (10). aPL is an autoantibody produced by B cells, and the activation of both B cells and B cell subsets contributes to the development of autoimmune diseases, including APS (11, 12). However, few studies have compared changes in peripheral blood CD19+B cells between APS patients and healthy controls.

An association between APS and disorders of both cell and humoral immunity has been reported, including abnormalities in the number and function of T lymphocyte subgroups (4). Although T helper 1 (Th1) and T helper 2 (Th2) cells previously attracted the most research interest, in recent years the focus has been on the imbalance between T helper 17 (Th17) and T regulatory (Treg) cell subsets (13–16), especially with respect to rheumatoid diseases and cancer. For APS patients, overactivated CD4+T cells activate B cells, which then produce high levels of autoantibodies (17, 18). In this complex process, Th17 cells play an indispensable role by secreting inflammatory cytokines such as interleukin-IL (IL-17), which stimulates antibody production (19, 20). Conversely, Treg cells, a subset of regulatory CD4+T cells, inhibit Th17 and B cells activation, and thus negatively regulate antibody production (21, 22). Research on Tregs has revolutionized our understanding of immune control mechanisms and immune-mediated diseases, thereby opening up many avenues for the development of a new generation of therapies based on the regulation of Treg cell expression and function. Imbalances in Th17 and Treg subsets in various autoimmune diseases have been demonstrated, including SLE (23), RA (24) and inflammatory bowel disease (IBD) (25, 26). However, Alvarez-Rodriguez L and colleagues found no difference in the number and function of Treg subset between PAPS patients and healthy controls, and no increase in the Th17/Treg ratio (27). However, studies on Th cell subsets in APS patients are scarce (28, 29).

Therefore, based on advancements in cell-detection technology and its clinical application for determining absolute cell numbers in lymphocyte subpopulations, we investigated differences between PAPS and SAPS patients and healthy controls with respect to peripheral blood lymphocytes, CD4+T cell subsets, and cytokines. Our aim was to elucidate the role of CD4+T cell subsets, especially Th17 and Treg cell subsets, and cytokines in the pathogenesis of APS.

## Materials and Methods

### Clinical Data

The 67 APS patients (19 males and 48 females) had an average age of 48.18 ± 14.931 years and were recruited from the Department of Rheumatology of the Second Hospital of Shanxi Medical University (Taiyuan, China) from June 2016 to June 2021.All patients were diagnosed according to Sapporo classification criteria, revised at the 11^th^ International Conference on Antiphospholipid Antibodies in 2006 (30). Patients with the following conditions were excluded: younger than 18 years of age; pregnancy; severe infections; and tumors. The 40 healthy controls of similar age and were recruited after a physical examination.

Patients with APS were divided into PAPS group (n=12) and SAPS group (n=55). The other diseases present in the latter group included SLE (n=23), SS (n=13), RA (n=2) and other autoimmune diseases (n=17). The retrospectively collected clinical and serological parameters included demographics and the following laboratory data: white blood cell (WBC) count, hemoglobin (Hb) level, platelet (PLT) count, lymphocyte (LY) count, erythrocyte sedimentation rate (ESR), C-reactive protein (CRP), aPL (including aCL-IgM and aβ2GPI-IgM), complement C3 and C4, coagulation function, blood lipid, absolute number and percentage of peripheral lymphocytes and T cell subsets, and cytokine level. All laboratory tests were conducted on blood freshly drawn from patients after a morning fast. The study was approved by the Ethics Committee of the Second Hospital of Shanxi Medical University [approval no (2019). YX No. (105)].

### Flow Cytometry Measurement of Absolute Number of Peripheral Lymphocytes and T Cell Subsets

To determine the absolute numbers and percentage of peripheral lymphocytes and CD4+T cell subsets, peripheral blood samples (2ml) were collected from patients and controls, and stored in tubes.

#### Absolute Numbers of T, B, Natural Killer, CD4+T, CD8+ T Cells in Peripheral Blood Lymphocytes

**Figure 1:**
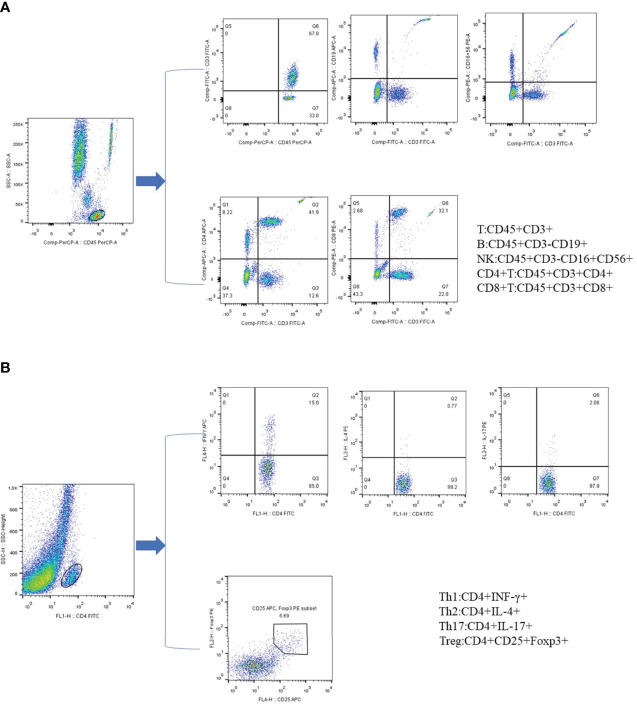
Phenotypic characterization of lymphocyte subpopulations by flow cytometry. **(A)** Representative flow cytometry analysis of peripheral lymphocytes. **(B)** Representative flow cytometry analysis of CD4+ T cell subsets. All dot plot analysis is of CD4+ gated lymphocyte.

Two BD Trucount tubes containing a known number of fluorescent microspheres were marked as A and B, and 50µL of peripheral blood sample were added to each one by reverse loading, followed by the addition 20µL of anti-CD3-FITC/CD-8PE/CD45PercP/CD4APC antibodies and CD3FITC/CD16+56-PE/CD45-PercP/CD19-APC antibodies, respectively. The mixture was shake-incubated at 25°C for 20 min in the dark, after which 450µL XFACS hemolysin was added; the incubated continued for 15min under the same conditions. For each sample, 15000 cells were collected (**Figure 1A**).

#### Detection of CD4+T Cell Subsets

First, 10 µL of phorbol myristate acetate working solution, 10 µL of ionomycin working solution, and 1 µL of GolgiStop were added to 80 µL of peripheral blood, and the mixture was incubated for 5h at 37°C in a 5% carbon dioxide atmosphere. The blood cells were then stained with anti-CD4-FITC antibody at 25°C in the dark, followed by the addition of 1 ml of freshly prepared fixation/permeabilization solution and a 30-min incubation at 4°C in the dark. Th1 cells were detected by anti-FITC-CD4 and anti-IFN-γ-APC, Th2 cells by anti-FITC-CD4 and anti-IL-4-PE, and Th17 cells by anti-human IL-17-PE. For the detection of Treg cells, 80 µl of blood was treated with CDC4-FITC antibody and CD25-APC antibody for 30 min in the dark at 25°C, followed by the addition of 1 ml of freshly prepared fixation/permeabilization mixed solution and incubation under the same conditions as described for the anti-FOXP3-PE testing of Treg cells (**Figure 1B**).

All immunofluorescence antibodies were purchased from BD Biosciences. All stained cells were assessed using flow cytometry (FACSCanto II; BD Biosciences. The data were analyzed using FlowJo V7.6.1 (Tree Star Inc., Ashland, OR, USA).

### Detection of Cytokine Levels by Cytometric Bead Array

First, 4 ml of venous blood was left to stand for 2 h at room temperature, after which the serum was separated from the sample and stored at −20°C. IL-2, IL-4, IL-6, IL-10, IL-17, interferon (IFN)-γ, and tumour necrosis factor (TNF)-α were detected by cell microsphere array (Jiangsu Saiqi Biotechnology Co., Ltd.). Captured data from standard and test specimens were transferred to the BD FCAP array software. The cytokine assay results are expressed as pg/ml.

### Statistical Analysis

Data with a normal distribution and homogeneity of variance are presented as the mean ± standard deviation and were compared between two groups using an independent-samples t-test. Data with a nonnormal distribution are presented as the median (interquartile range) and were compared between two groups using the Mann-Whitney U test. Categorical variables are presented as frequencies and were compared using the chi-squared test. Correlation analysis was performed using Pearson’s or Spearman’s correlation. In all analyses, a p-value < 0.05 was considered to indicate statistical significance. All statistical analyses were performed using SPSS 23.0 (SPSS Inc, Chicago, IL, USA) and GraphPad Prism software (version 9.0; GraphPad Software Inc., La Jolla, CA, USA).

## Results

### Clinical and Demographic Features

The main demographics feature, laboratory data, disease characteristics, traditional cardiovascular risk factors and current medication of 67 APS patients (12 with PAPS and 55 with SAPS) are presented in **Table 1**. The most common co-existing autoimmune disease in SAPS patients was SLE, accounting for 41.82%, followed by SS (23.61%), while RA only accounted for less than 4%. Among the traditional cardiovascular risk factors, SAPS and PAPS patients did not significantly differ in serum lipid levels, hypertension, diabetes, or family history of cardiovascular disease. The frequency of hormone use in SAPS patients was 85.45%, which was significantly higher than that in PAPS patients, while the frequency of statin use was higher in the latter. The frequency of anticoagulant use was nearly the same between the PAPS and SAPS groups (58.33% and 54.55%, respectively).

**Table 1:** Characteristics of PAPS group and SAPS group.

	PAPS	SAPS	P-value
Demographics			
Age (year) ^a^	50.75 ± 13.34	47.62 ± 15.31	0.515
Male, n (%)	4 (33.33%)	15 (27.27%)	0.478
Female, n (%)	8 (66.67%)	40 (72.73%)	
BMI (Kg/m^2^) ^a^	25.20 ± 4.82	24.00 ± 3.16	0.304
Disease duration (month) ^b^	121.00 (39.50-132.00)	40.00 (13.00-108.00)	0.177
Laboratory characteristics			
ESR (mm/h) ^b^	31.00 (14.00-40.00)	30.00 (10.50-66.00)	0.776
CRP (mg/ml) ^b^	3.13 (2.32-5.98)	3.73 (3.12-10.28)	0.297
WBC (*10^9/L) ^b^	6.73 (5.15-7.56)	5.61 (3.66-8.37)	0.475
Hb (g/L) ^a^	124.92 ± 21.48	114.61 ± 28.62	0.245
PLT (*10^9/L) ^b^	183.00 (56.00-218.00)	120.00 (82.00-237.75)	0.758
LY (*10^9/L) ^b^	1.59 (1.11-1.67)	1.07 (0.77-1.42)	0.019*
aCL (MPLU/mL) ^b^	63.05 (14.30-120.00)	32.2 (13.86-120.00)	0.771
aβ2GPI (AU/ml) ^b^	70.53 (57.74-128.91)	68.95 (24.38-185.00)	0.662
C3 (g/L) ^a^	0.71 ± 0.99	0.65 ± 0.25	0.507
C4 (g/L) ^b^	0.17 (0.11-0.22)	0.12 (0.08-0.19)	0.184
Level of blood coagulation			
Fibrinogen (mmol/L) ^b^	2.80 (2.59-3.15)	2.75 (2.30-3.52)	0.993
APTT(s) ^b^	34.40 (30.05-50.55)	39.00 (29.00-56.35)	0.705
PT(s) ^b^	14.80 (14.18-15.18)	15.50 (14.10-16.80)	0.189
D-Dimer (ug/L) ^b^	92.00 (81.00-286.00)	215.00 (60.25-595.75)	0.530
Serum lipid level			
Triglycerides (mmol/L) ^b^	0.98 (0.76-1.48)	1.36 (1.03-1.66)	0.199
Total cholesterol (mmol/L) ^b^	3.72 (3.11-4.41)	3.64 (3.01-4.80)	0.825
LDL (mmol/L) ^b^	2.10 (1.73-2.47)	1.94 (1.40-2.70)	0.666
HDL (mmol/L) ^b^	0.99 (0.96-1.56)	1.17 (0.90-1.54)	0.962
Clinical measures			
Thrombocytopenia, n (%)	6 (50.00%)	20 (36.36%)	0.380
Thrombosis, n (%)	7 (58.33%)	29 (52.73%)	0.724
obstetric manifestations, n/female (%)	6/7 (85.71%)	14/41 (34.15%)	0.005**
Cardiovascular risk factors			
Smoking, n (%)	4 (33.33%)	7 (12.73%)	0.081
Drinking, n (%)	5 (41.67%)	7 (12.73%)	0.018*
Hypertension, n (%)	4 (33.33%)	12 (21.82%)	0.397
Diabetes, n (%)	2 (16.67%)	8 (14.55%)	0.852
Family history of CVD, n (%)	2 (16.67%)	11 (20.00%)	0.791
Co-existing autoimmune disease			
SLE, n (%)	–	23 (41.82%)	–
SS, n (%)	–	13 (23.61%)	–
RA, n (%)	–	2 (3.64%)	–
Other autoimmune disease, n (%)	–	17 (30.93%)	–
Current use of medication			
NSAIDs, n (%)	4 (33.33%)	27 (49.09%)	0.321
DMARDs, n (%)	6 (50.00%)	27 (49.09%)	0.999
Hormone, n (%)	5 (41.67%)	47 (85.45%)	0.003**
Anticoagulant, n (%)	7 (58.33%)	30 (54.55%)	0.998
Statins, n (%)	5 (41.67%)	7 (12.73%)	0.032*

a Results are expressed as the mean ± standard deviation.

b Results are expressed as the median and 25th and 75th percentiles.

The independent-samples t-test was used for analysis of quantitative variables with normal distributions. Mann-Whitney U test was used for analysis of quantitative variables with a non-normal distribution. Chi-square test was used for categorical variables.

PAPS, primary antiphospholipid syndrome; SAPS, secondary antiphospholipid syndrome; BMI, body mass index; ESR, erythrocyte sedimentation rate; CRP,C-reactive protein; WBC, white blood cell; Hb, hemoglobin; PLT, platelet; LY, lymphocyte; aCL, anticardiolipin antibody; aβ2GPI, anti-β2 glycoprotein-I; C3, complement C3; C4, complement C4; APTT, activated partial thromboplastin time; PT, prothrombin time; LDL, low density lipoprotein; HDL, high density lipoprotein; CVD, cardiovascular diseases; SLE, systemic lupus erythematosus; SS, sjogren’s syndrome; RA, rheumatoid arthritis; NSAIDs, nonsteroidal antiinflammatory drugs; DMARDs, disease-modifying antirheumatic drugs. *P<0.05, **P<0.01.

A comparison of immune-related indicators showed significantly lower peripheral blood lymphocyte levels in SAPS than in PAPS patients. Laboratory findings, including ESR and CRP, which roughly represent disease activity, and clinical measures, such as thrombocytopenia and thrombosis, did not differ significantly between the two groups. While PAPS patients had higher titres of aCL and aβ2GPI antibodies, as well as higher complement C3 and C4 levels, than SAPS patients, none of the differences were statistically significant.

### Decreased Peripheral Treg Cells and an Increased Th17/Treg Ratio in PAPS and SAPS Patients

**Figure 2:**
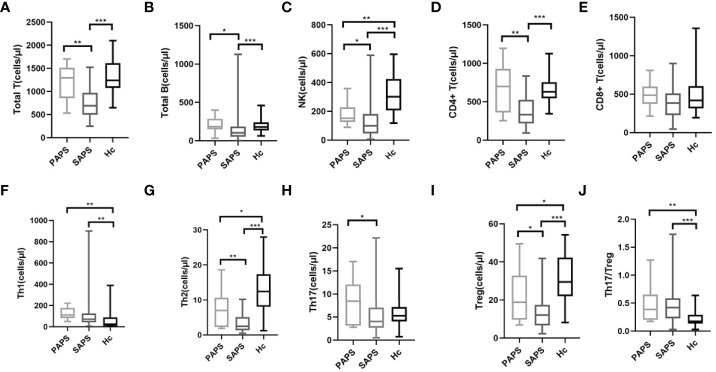
Comparison of lymphocyte absolute values and CD4+ T cell subsets in PAPS group (n=12), SAPS group (n=55) and healthy control group (n=40) (∗p < 0.05, ∗∗p < 0.01, and ∗∗∗p < 0.001). Compared with the healthy control group, the differences in Treg cell subset and Th17/Treg value were statistically significant, regardless of PAPS group or SAPS group. **(A–E)** represent the differences in the absolute value of lymphocyte in three groups (corresponding to Total T, Total B, NK, CD4+T, CD8+T,respectively). **(F–J)** means the differences in absolute value of Th subsets of three groups (corresponding to Th1, Th2, Th17, Treg, Th17/Treg,respectively).

The levels of peripheral blood lymphocyte and CD4+T cell subsets, including Th1, Th2, Th17, and Treg cells, were compared among the PAPS, SAPS, and healthy control groups. For peripheral blood lymphocyte subsets, the SAPS group differed significantly from the healthy controls with respect to T cells [689.26 (494.89–971.96) vs. 1239.00 (1075.25–1611.75), p<0.001], B cells [104.69 (51.83–185.26) vs. 177.50 (135.25–240.50), p<0.001], NK cells [98.97 (46.35–181.25) vs. 300.00 (206.50–424.00), p<0.001] and CD4+T cells [330.16 (218.34–524.43) vs. 628.50 (545.50–755.75), p>0.001]. However, in comparisons of PAPS patients and healthy controls, the only difference was a decrease in NK cells [151.30 (124.72–227.42) vs. 300.00 (206.50–424.00), p=0.002] (**Supplementary Table 1**; **Figures 2A–D**). In a comparison of PAPS and SAPS patients, the former had significantly higher counts of T cells [1295.41 (854.12–1511.96) vs. 689.26 (494.89–971.96), p=0.001], B cells [184.44 (148.23–287.02) vs. 104.69 (51.83–185.26), p=0.012], NK cells [151.30 (124.72–227.42) vs. 98.97 (46.35–181.25), p=0.023], and CD4+T cells [698.34 (361.24–927.90) vs. 330.16 (218.34–524.43), p=0.002]. These results suggest immune cell disorders in PAPS and SAPS patients.

It is worth noting that PAPS and SAPS groups showed the same trend in CD4+T cell subsets compared with healthy controls (**Supplementary Table 2**; **Figures 2F–J**). However, in both the PAPS and SAPS groups, an increase in Th1 cells [111.50 (81.15–176.29) vs. 23.47 (6.78–88.77), p=0.002 and 71.43 (41.01–124.43) vs. 23.47 (6.78–88.77), p=0.001, respectively] and decrease in Th2 cells [6.97 (2.27–10.63) vs.12.43 (8.07–17.38), p=0.037 and 2.49 (1.36–5.12) vs. 12.43 (8.07–17.38), p<0.001, respectively] were detected. Furthermore, in the PAPS and SAPS groups, Treg cells were lower [18.77 (9.55–32.86) vs. 29.53 (22.02–42.20), p=0.031 and 12.01 (6.55–17.49) vs. 29.53 (22.02–42.20), p<0.001, respectively], and the Th17/Treg ratio was higher [0.39 (0.20–0.66) vs. 0.17 (0.13–0.29), p=0.001 and 0.42 (0.23–0.59) vs. 0.17 (0.13–0.29), p<0.001, respectively], compared to the healthy control group, while Th17 cells did not significantly differ [8.42 (3.12–12.09) vs. 5.26 (4.05–7.17), p=0.184 and 4.00 (2.69–7.06) vs. 5.26 (4.05–7.17), p=0.072, respectively]. This implies that, compared with the healthy population, APS patients have an imbalance in peripheral Th17 and Treg cells, most likely attributable to a decrease in the absolute number of Treg cells. CD4+T cell subsets were also compared between the PAPS and SAPS groups. The results showed that PAPS patients had significantly higher levels of Th2 cells [6.97 (2.27–10.36) vs. 2.46 (1.36–5.12), p=0.006], Th17 cells [8.42 (3.12–12.09) vs. 4.00 (2.69–7.06), p=0.042] and Treg cells [18.77 (9.55–32.86) vs. 12.01 (6.55–17.49), p=0.020] (**Supplementary Table 2**; **Figures 2G–I**), suggesting immune cell differences between PAPS and SAPS.

### Significantly Higher Levels of Several Cytokines in APS Patients Than in Healthy Controls and a Negative Correlation With aCL Titre

Cytokine levels (including IL-2,4,6,10, 17, IFN-γ, and TNF-α) were also compared between PAPS and SAPS patients. Only the IL-10 level differed; it was significantly lower in PAPS than SAPS patients [4.71 (2.8–5.55) vs. 5.69 (3.94-8.38), p=0.032] (**Supplementary Table 3**). Regarding differences in cytokine levels between the combined PAPS and SAPS (APS) group and healthy controls, the former group showed significantly higher levels of all cytokines, including IL-2 [2.54 (1.80–3.47) vs. 1.86 (1.44–2.38), p=0.008], IL-4 [2.87 (1.82–4.58) vs. 1.82 (1.43–2.31), p<0.001], IL-6 [6.49 (4.87–11.27) vs. 3.92(2.45–6.07), p<0.001], IL-10 [5.20 (3.62–7.86) vs. 2.99 (2.40–4.37), p<0.001], IL-17 [7.69 (3.07–17.61) vs. 4.80 (2.51–6.01), p=0.005], IFN-γ [4.20 (2.61–6.91) vs. 2.60 (2.13–3.56), p=0.002] and TNF-α [3.34 (1.93–5.88) vs. 2.02 (1.48–2.46), p<0.001] (**Table 2**).

**Table 2:** Cytokine levels (pg/ml) in APS group and heathy control group.

Cytokine levels (pg/ml)	APS group (n=37)	Heathy control group (n=31) ^a^	P-value
IL-2	2.54 (1.80-3.47)	1.86 (1.44-2.38)	0.008**
IL-4	2.87 (1.82-4.58)	1.82 (1.43-2.31)	<0.001***
IL-6	6.49 (4.78-11.27)	3.92 (2.45-6.07)	<0.001***
IL-10	5.20 (3.62-7.86)	2.99 (2.40-4.37)	<0.001***
IL-17	7.69 (3.07-17.61)	4.80 (2.51-6.01)	0.005**
IFN-γ	4.20 (2.61-6.91)	2.60 (2.13-3.56)	0.002**
TNF-α	3.34 (1.93-5.88)	2.02 (1.48-2.46)	<0.001***

^a^means that 9 sets of data are lost.

Results are expressed as the median and 25th and 75th percentiles.

Statistics: Mann-Whitney U test.

APS, antiphospholipid syndrome; IL-2, interleukin-2; IL-4, interleukin-4; IL-6, interleukin-6; IL-10, interleukin-10; IL-17, interleukin-17; INF-γ, interferon-γ; TNF-α, tumor necrosis factor-α. *P<0.05, **P<0.01, ***P<0.001.

**Figure 3:**
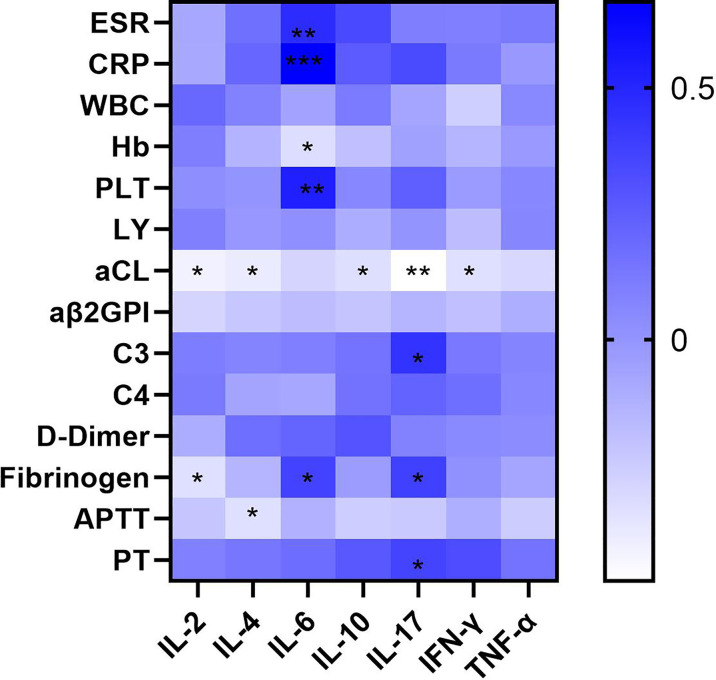
Heatmap of correlation of the serum cytokine levels of a variety of cytokines with clinical and laboratory characteristics of APS patients (∗p < 0.05, ∗∗p < 0.01, and ∗∗∗p < 0.001). The aCL titer was negatively correlated with many cytokines including IL-2, IL-4, IL-10, IL-17, INF-γ, and IL-6 was positively correlated with ESR and CRP.

An analysis of the correlations between cytokines and laboratory data in APS patients (**Supplementary Tables 4, 5**) showed that IL-2 (r = −0.420, p=0.010), IL-4 (r = −0.392, p=0.016), IL-10 (r = −0.331, p=-0.046), IL-17 (r = −0.479, p=0.006), and IFN-γ (r = −0.339, p=0.040) correlated negatively with the titres of the aPL aCL (**Figure 3**). Conversely, IL-6 correlated significantly and positively with ESR (r = 0.469, p=0.004) and CRP (r = 0.670, p<0.001), which provide a rough indication of disease activity. IL-17 correlated positively with the complement C3 (r = -0.440, p=0.025) and fibrinogen levels (r = 0.380, p=0.032), and prothrombin time (r = 0.360, p=0.043). Other cytokines associated with fibrinogen included IL-2 (r = −0.336, p=0.042) and IL-6 (r = 0.372, p=0.023). IL-4 correlated negatively with the activated partial thromboplastin time (r = -0.337, p=0.042) and IL-6 correlated positively with the platelet count (r = 0.530, p<0.001). These results indicate that abnormalities in many cytokines are involved in the pathogenesis of APS.

### aCL and aβ2GPI Titres Correlate Positively With Th17/Treg Values in PAPS Patients

**Figure 4:**
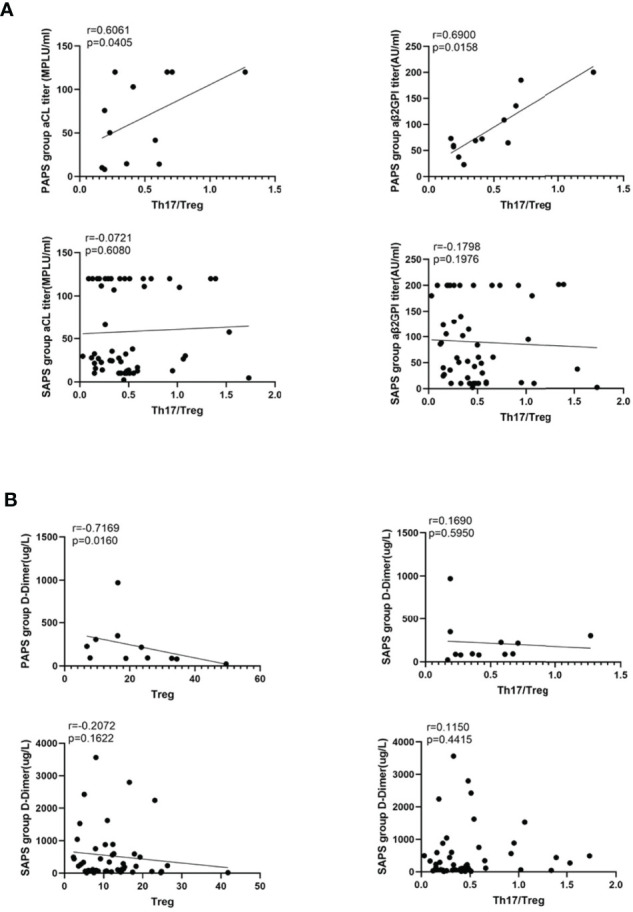
**(A)** The correlation analysis between the value of Th17/Treg and the titer of aCL and aβ2GPI in PAPS group and SAPS group, respectively. **(B)** shows the correlation between D-Dimer and Treg cell subset, Th17/Treg value respectively in PAPS and SAPS groups. The linear regression line of the interpolated 95% confidence interval curve (broken line) is shown.

A simple linear regression analysis was used to determine the correlations of aCL and aβ2GPI titres with Th17/Treg values in the PAPS and SAPS groups. As shown in **Figure 4A**, neither aCL (r = −0.0721, p=0.6080) nor aβ2GPI (r = −0.1798, p=0.1976) was associated with the Th17/Treg ratio in the SAPS group, whereas the titres of both antibodies correlated positively with the Th17/Treg ratio in the PAPS group (aCL: r = 0.6061, p=0.0405; aβ2GPI: r = 0.6900, p=0.0158). Thus, an imbalance in Th17 and Treg subsets may contribute to autoantibody production in PAPS patients.

### The D-Dimer Level Correlates Negatively With Treg Cell Subsets in PAPS Patients

Arterial and venous thrombosis are major threats to APS patients. Since D-dimer is a sensitive marker of thrombosis, we investigated its correlation with Treg cells and the Th17/Treg ratio in the PAPS and SAPS groups (**Figure 4B**). In PAPS patients, D-dimer levels correlated negatively with Treg cell subsets (r = -0.7169, p=0.0160) but were not related to the Th17/Treg ratio (r = 0.1690. p=0.5950). However, in the SAPS group, D-dimer levels were not associated with either Treg cells or the Th17/Treg ratio (r = −0.2072, p=0.1622 and r = 0.1150, p=0.4415, respectively). These results suggest a link between Tregs and thrombosis in PAPS, but not SAPS, patients.

### Elevated Serum Complement C3 and C4 Levels in ANA-Negative SAPS Patients

**Figure 5:**
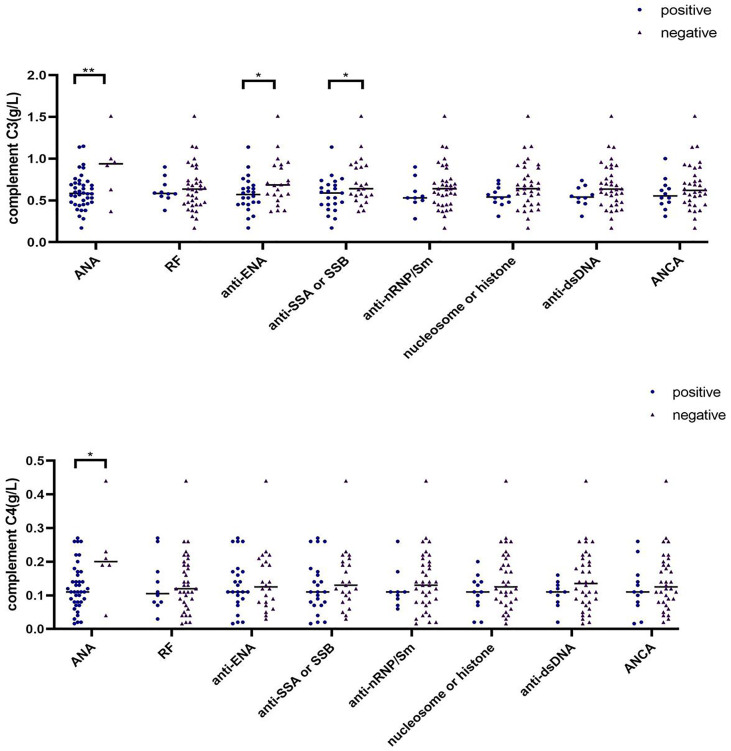
Differences in complement C3 and C4 levels among different antibody subgroups in SAPS group (∗p < 0.05, ∗∗p < 0.01).

In addition to antiphospholipid antibody, other autoantibodies detected in the SAPS group mainly included antinuclear antibody (ANA, n=46), rheumatoid factor (RF, n=12), anti-extractable nucler antigen (anti-ENA, n=27), and anti-SSA or SSB (n=25), anti-nRNP/Sm (n=12), nucleosome or histone (n=12), anti-double-stranded DNA (anti-dsDNA, n=10) and anti-neutrophil cytoplasmic antibody (ANCA, n=16). In some patients, two or more autoantibodies were detected. We thus divided patients into different groups according to their autoantibody positivity or negativity, and compared the difference of C3 and C4 between different groups (**Supplementary Tables 6, 7**; **Figure 5**). ANA-negative patients had higher serum C3 and C4 levels than ANA-positive patients (p=0.007 and p=0.047, respectively), and anti-ENA positive patients had lower serum C3 levels that anti-ENA-negative patients, similar to the findings for anti-SSA or SSB. However, serum C4 levels were not affected by autoantibodies other than ANA.

## Discussion

This study showed significant decreases in NK cells in PAPS patients, and in T, B, and CD4+T cells in SAPS patients, compared to a healthy control group. Among CD4+T cell subsets, similar trends were demonstrated in PAPS and SAPS patients, including increased Th1 cells and decreased Th2 cells. In addition, a decrease in Treg cells accounted for the increase in the Th17/Treg ratio in all APS patients compared to the healthy controls, but Th17 and Treg cell subsets were higher in PAPS than SAPS patients. Among APS patients, the levels of many cytokines were higher than in healthy controls and were associated with the aCL titre. Correlation analysis suggested that, for PAPS patients, the titres of two autoantibodies, aCL and aβ2GPI, correlated positively with the Th17/Treg ratio and negatively with both D-dimer levels and Treg subsets. In the SAPS group, serum complement C3 and C4 levels were elevated in ANA-negative patients. Taken together, these findings implicate Th17 and Treg cell subsets in the pathogenesis of APS, and in the different inflammatory profiles of PAPS and SAPS patients. As such, our study sheds light on the complex mechanisms of APS, which may improve its assessment and management.

Previous studies examining CD19+B cell numbers in the peripheral blood of APS patients yielded contradictory results. Dal Ben et al. reported decreases in the number of B cells in both PAPS (31) and APS secondary to SLE (32). Our results were consistent with those studies, as we determined a reduction in the number of peripheral blood B cells in PAPS and in SAPS patients. However, a 2018 study by Alvarez-Rodriguez L et al. (33) found no difference in the number and percentage of B cells in PAPS patients, with or without venous thrombosis, compared to healthy controls. The discrepancies may be related to the study population, the use of baseline drugs, and the timing of the study. The latter study also showed that PAPS patients had a lower proportion of naïve B cells and higher number of non-switched memory B cells, in contrast to the results of Simonin et al. (11). The difference in distribution of CD19+B cells in the peripheral blood of PAPS and SPAS patients is the current focus of our team’s research.

The imbalances in lymphocyte and T cell subsets in APS patients, and their role in APS pathogenesis and disease surveillance, have attracted increasing research attention in recent years (34, 35). In the above-mentioned study of Alvarez-Rodriguez et al. (27), it was concluded that Th cells did not play a direct role in PAPS; however, only PAPS, SLE, and healthy controls were compared; the study did not include SAPS patients. Simonin L et al. (11) investigated 22 PAPS patients with venous thromboembolism (VTE) and found disturbances in T and B cell homeostasis; however, they did not assess the atatus of Th subsets. A strength of our study was its full analysis of lymphocyte, Th subsets and cytokines in both PAPS and SAPS patients, and the comparison with a healthy control group. We were thus able to show reductions in of T, B, NK, and CD+T cells in PAPS and SAPS patients compared to healthy controls. While this result might be attributed to the use of immunosuppressants by APS patients, the finding that Treg cells were reduced in the PAPS and SAPS groups, whereas the Th17/Treg ratio was elevated, instead points to direct involvement of an imbalance in Th17 and Treg cell subsets in the pathogenesis of APS. This was also the conclusion of Jakiela et al. (29), but the main focus of their work was the effect of aPL-IgG titres on Th cell subsets. Treg and Th17 cells have a common progenitor and their differentiation, development and expression are regulated by transforming growth factor-β-mediated signaling (16, 36) as well as IL-2 (37–39). Th17 cells, a subset of pro-inflammatory cells, produce IL-17A, IL-17F, and IL-22 and participate in signal induction and dissemination (40, 41), whereas Treg cells down-regulate the activation and proliferation of self-reactive T cells and maintain immune tolerance (42, 43). Treg cells express the α chain of the IL-2 receptor on their surface (44); in response to IL-2 binding, they secrete negative immune regulatory cytokines such as IL-6 and IL-10 (45). Our study showed higher levels of these cytokines in APS patients than healthy controls, and their association with the aCL titre. We therefore speculate that, in APS patients, there is an imbalance in the number of Th17 and Treg subsets, as well as an impairment of the function of these cells.

Few studies have examined the differences between PAPS and SAPS patients. A cohort study of 148 APS patients in Egypt found that SAPS patients were more prone to systemic manifestations, venous thrombosis, cutaneous vasculitis, thrombocytopenia and hemolytic anemia (46). The authors also suggested different disease presentations depending on ethnicity. Unfortunately, our study does not reach the same conclusion. Other studies have showed that patients with SAPS are more likely to develop more severe diffuse alveolar hemorrhage (DAH) and non-infectious endocarditis, known as Libman-Sacks (LS) endocarditis, especially in APS secondary to SLE (47, 48). Only one other study reported differences in immune function between PAPS and SAPS patients, but the lower serum C3 and C4 levels in PAPS patients (9) were inconsistent with our results. Further studies are needed to determine the differences in immune function between PAPS and SAPS. Our analysis of peripheral blood lymphocyte and CD4+T cell subsets in PAPS and SAPS patients showed that the levels of T, B, NK, Th2, Th17 and Treg cells in PAPS were higher than those in SAPS, which may be related to the fact that most patients with SAPS received immunosuppressive therapy including DMARDs. The cytokine analysis indicated that the IL-10 level in PAPS patients was lower than that in SAPS patients, which may indicate that the immune regulation ability of PAPS patients is slightly weaker.

Recurrent thrombosis is an important feature of APS and complicates its management. APS patients are prone to a vicious cycle of thrombotic events (of which 50% are lower limb deep vein thromboses, with a high risk of pulmonary embolism) (49) that result in thrombocytopenia, which in turn increases the risk of thrombosis (50). Most APS patients are therefore prescribed anticoagulants, such as vitamin K antagonists and low molecular weight heparin. However, thrombus in APS is a complex process, involving oxidation of the domain 1 Arg 39 Arg 43 epitope and cross-linking of several surface receptors (51), such that anticoagulation therapy should be accompanied by immunological therapy. A 2021 study (52) suggested that Treg populations in the thrombus form stromal acid and cysteine-rich proteins (SPARC) to promote clot breakdown, by recruiting monocytes and promoting fibrinolytic activity to disrupt the fibrin network. According to this scenario, the balance of Treg cells plays an important role in thrombolysis and the prevention of recurrence. Our study also identified a negative correlation between D-dimer levels and Treg cell subsets in PAPS patients. As D-dimer is a sensitive indicator of hypercoagulability, this relationship would seem to implicate immune cells, especially Treg cell subsets, in thrombogenesis characteristic of APS, although the specific mechanism remains to be explored. In our study, due to the small number of blood samples in the PAPS group, we used logistics regression analysis to examine the correlation of clinical indicators with the presence of thrombosis in PAPS, but the result was not significant (data not shown).

ANA positive is mostly associated with adverse pregnancy outcomes (APO) in OAPS patients, as demonstrated in animal models (53) and clinical studies (54). In addition, high levels of C4a and C3b deposition were found in the placenta of OAPS women. Complement activation may be a key process in aPL-related thrombosis and fetal loss (55), and hypocomplement was shown to be an independent predictor of lower neonatal birth weight and premature delivery (56). A real-world prospective study concluded that high serum C3 levels in early pregnancy protect against obstetric complications in OAPS, whereas ANA titres > 1:160 are an independent risk factor (57). Similarly, our study identified higher serum C3 and C4 levels in ANA-negative SAPS patients. These results suggest that ANA also plays a role in the aPL-mediated activation of complement, leading to APO and thrombosis.

Although we are aware of the limitation regarding the possible effect of different therapies in SAPS, which were not considered as confounding factors. Patients with SAPS were treated with steroids, antimalarials, antiplatelet, and/or anticoagulant therapies. The effects of glucocorticoids on Th subsets, especially Th1 and Th17, have been studied and demonstrated (58, 59). And the immunosuppressive effects of hydroxychlorochin, a classic antimalarial drug, is well-known. Indeed, we did not find any effect of anticoagulants on lymphocytes. There is only one study (60) showing that prasugrel had an inhibitory effect on effector Th cells *in vitro*, which were induced to develop an inflammatory phenotype by the addition of platelets. Our patients did not receive prasugrel. These further confirms our findings that Th17/Treg responses are stronger in SPAS compared to healthy individuals, despite the inhibitory effect of treatment.

Despite its novel findings, our study also had several limitations. Frist, the sample size was small, due to the limited number of patients with a definitive diagnosis of APS, especially PAPS. Second, as it was a retrospective study, peripheral blood samples did not suffice for *in vitro* experiments. Third, direct evidence of a role of an imbalance between Th17 and Treg subsets in the pathogenesis of APS awaits confirmation by appropriate animal models. Fourth, the baseline medications in our APS patients differed, and neither ESR nor CRP was an accurate indicator of disease activity. Finally, the timing of thrombosis was not reported. Some of these issues may have had an important impact on peripheral blood lymphocyte subsets and cytokine levels.

Although it could not establish the precise mechanism by which Th17 and Treg cells contribute to APS, our study suggested that the increased Th17/Treg ratio resulting from decreased Treg cells is related to the occurrence of APS. If confirmed in further studies, this would support the use of immunomodulatory rather than immunosuppressive therapy in APS. Further studies are needed to examine the participation of Treg cells and Treg-derived cytokines in APS. In addition, we found immunological differences between PAPS and SAPS patients, but this may be related to the majority of SAPS patients receiving immunosuppressive therapy. More differences in immunity between the two are worth further exploration.

## Data Availability Statement

The raw data supporting the conclusions of this article will be made available by the authors, without undue reservation.

## Ethics Statement

The studies involving human participants were reviewed and approved by Ethics Committee of the Second Hospital of Shanxi Medical University. The patients/participants provided their written informed consent to participate in this study.

## Author Contributions

HY performed the data analyses and wrote the manuscript. BL participated in the collection of samples and clinical data. RS participated in the performance of the research and statistical analysis. CG and XL participated in the study design and revising of the manuscript. CW provided intellectual input and supervision throughout the study and made a substantial contribution to manuscript drafting. All authors contributed to the article and approved the submitted version.

## Funding

This work was supported by National Natural Science Foundation of China (No. 81971543, No. 81471618); Key Research and Development (R&D) Projects of Shanxi Province (201803D31119).

## Conflict of Interest

The authors declare that the research was conducted in the absence of any commercial or financial relationships that could be construed as a potential conflict of interest.

## Publisher’s Note

All claims expressed in this article are solely those of the authors and do not necessarily represent those of their affiliated organizations, or those of the publisher, the editors and the reviewers. Any product that may be evaluated in this article, or claim that may be made by its manufacturer, is not guaranteed or endorsed by the publisher.
