# Erratum: Preliminary Study on the Imbalance Between Th17 and Regulatory T Cells in Antiphospholipid Syndrome

**DOI:** 10.3389/fimmu.2022.971769

**Published:** 2022-06-23

**Authors:** Frontiers Production Office

**Affiliations:** Frontiers Media SA, Lausanne, Switzerland

**Keywords:** primary antiphospholipid syndrome (PAPS), secondary antiphospholipid syndrome (SAPS), T helper 17 (Th17) cells, regulatory T (Treg) cells, cytokines

Due to a production error, there was a mistake in **Table 2** as published. The four “>“ in the column of “p-value” in **Table 2** should all be “<“. The corrected **Table 2** appears below. The publisher apologizes for this mistake.

**Table 2:** Cytokine levels (pg/ml) in APS group and heathy control group.

Cytokine levels (pg/ml)	APS group (n=37)	Heathy control group (n=31) ^a^	P-value
IL-2	2.54 (1.80-3.47)	1.86 (1.44-2.38)	0.008**
IL-4	2.87 (1.82-4.58)	1.82 (1.43-2.31)	<0.001***
IL-6	6.49 (4.78-11.27)	3.92 (2.45-6.07)	<0.001***
IL-10	5.20 (3.62-7.86)	2.99 (2.40-4.37)	<0.001***
IL-17	7.69 (3.07-17.61)	4.80 (2.51-6.01)	0.005**
IFN-γ	4.20 (2.61-6.91)	2.60 (2.13-3.56)	0.002**
TNF-α	3.34 (1.93-5.88)	2.02 (1.48-2.46)	<0.001***

^a^means that 9 sets of data are lost.

Results are expressed as the median and 25th and 75th percentiles.

Statistics: Mann-Whitney U test.

APS: antiphospholipid syndrome; IL-2: interleukin-2; IL-4: interleukin-4; IL-6: interleukin-6; IL-10: interleukin-10; IL-17: interleukin-17; INF-γ: interferon-γ; TNF-α: tumor necrosis factor-α. *P<0.05, **P<0.01, ***P<0.001.

The original version of this article has been updated.

