# Supplementary material for: Preliminary Study on the Imbalance Between Th17 and Regulatory T Cells in Antiphospholipid Syndrome
Source: Front Immunol. 2022 May 6;13:873644. doi: 10.3389/fimmu.2022.873644 (PMC9121099; doi:10.3389/fimmu.2022.873644)
Supplement: Supplementary file 1 [file Table_1.docx]

Supplementary Material

# Supplementary Tables

| **Table 1:** Absolute counts and proportions of lymphocyte in the peripheral blood in PAPS group (A), SAPS group (B) and healthy control group (C). | | | | | | |
| --- | --- | --- | --- | --- | --- | --- |
| Cell count  Cells/μL | PAPS group (A) | SAPS group (B) | Heathy control group (C) | P-value  A vs. B | P-value  A vs. C | P-value  B vs. C |
| Total T | 1295.41  (854.12-1511.96) | 689.26  (494.89-971.96) | 1239.00  (1075.25-1611.75) | 0.001** | 0.811 | ＜0.001*** |
| T% | 73.98  (66.75-81.84) | 75.03  (65.70-79.22) | 70.00  (64.00-75.00) | 0.901 | 0.324 | 0.147 |
| Total B | 184.44  (148.23-287.02) | 104.69  (51.83-185.26) | 177.50  (135.25-240.50) | 0.012* | 0.397 | ＜0.001*** |
| B% | 11.91  (7.70-16.05) | 11.00  (6.03-20.31) | 10.00  (8.00-13.00) | 0.782 | 0.251 | 0.428 |
| NK | 151.30  (124.72-227.42) | 98.97  (46.35-181.25) | 300.00  (206.50-424.00) | 0.023* | 0.002** | ＜0.001*** |
| NK% | 10.18  (8.03-16.90) | 10.06  (5.64-18.24) | 16.5  (12.00-21.75) | 0.702 | 0.031* | 0.002** |
| CD4+ | 698.34  (361.24-927.90) | 330.16  (218.34-524.43) | 628.50  (545.50-755.75) | 0.002** | 0.965 | ＜0.001*** |
| CD4+% | 37.71  (30.19-47.60) | 34.03  (24.21-39.51) | 37.00  (31.25-40.00) | 0.197 | 0.598 | 0.108 |
| CD8+ | 487.01  (373.04-599.68) | 383.40  (227.93-509.75) | 418.00  (313.75-606.00) | 0.070 | 0.447 | 0.088 |
| CD8+% | 30.68  (27.51-35.02) | 34.78  (26.87-43.93) | 24.00  (19.25-29.00) | 0.178 | 0.022* | ＜0.001*** |
| CD4+T/CD8+T | 1.23  (0.77-1.50) | 1.00  (0.60-1.50) | 1.55  (1.24-2.06) | 0.241 | 0.106 | ＜0.001*** |
| Results are expressed as the median and 25th and 75th percentiles.   Statistics: Kruskal-Wallis test.   PAPS: primary antiphospholipid syndrome; SAPS: secondary antiphospholipid syndrome; Hc: healthy control. Total T: total T cells; Total B: total B cells; NK: natural killer T cells; CD4+: CD4+ T cells; CD8+: CD8+ T cell. *P<0.05, **P<0.01, ***P<0.001 | | | | | | |

| **Table 2:** Absolute counts and proportion of CD4+T cells in the peripheral blood in PAPS group (A), SAPS group (B) and healthy control group (C). | | | | | | |
| --- | --- | --- | --- | --- | --- | --- |
| Cell count  Cells/μL | PAPS group (A) | SAPS group (B) | Heathy control group (C) | P-value  A vs. B | P-value  A vs. C | P-value  B vs. C |
| Th1 | 111.50  (81.15-176.29) | 71.43  (41.01-124.34) | 23.47  (6.78-88.77) | 0.060 | 0.002** | 0.001** |
| Th1% | 19.80  (16.83-26.39) | 24.81  (13.76-33.81) | 3.55  (1.05-15.02) | 0.592 | ＜0.001*** | ＜0.001*** |
| Th2 | 6.97  (2.27-10.63) | 2.46  (1.36-5.12) | 12.43  (8.07-17.38) | 0.006** | 0.037* | ＜0.001*** |
| Th2% | 1.03  (0.72-1.73) | 0.76  (0.50-1.12) | 1.87  (1.05-2.63) | 0.053 | 0.025* | ＜0.001*** |
| Th17 | 8.42  (3.12-12.09) | 4.00  (2.69-7.06) | 5.26  (4.05-7.17) | 0.042* | 0.184 | 0.072 |
| Th17% | 1.12  (0.99-1.85) | 1.18  (0.73-2.13) | 0.81  (0.52-1.25) | 0.913 | 0.025* | 0.008** |
| Treg | 18.77  (9.55-32.86) | 12.01  (6.55-17.49) | 29.53  (22.02-42.20) | 0.020* | 0.031* | ＜0.001*** |
| Treg% | 3.43  (2.50-4.82) | 2.98  (2.36-3.87) | 4.52  (3.70-6.01) | 0.627 | 0.059 | ＜0.001*** |
| Th1/Th2 | 22.20  (14.39-28.02) | 31.78  (14.40-50.99) | 1.81  (0.78-6.47) | 0.098 | ＜0.001*** | ＜0.001*** |
| Th17/Treg | 0.39  (0.20-0.66) | 0.42  (0.23-0.59) | 0.17  (0.13-0.29) | 0.973 | 0.001** | ＜0.001*** |
| Results are expressed as the median and 25th and 75th percentiles.   Statistics: Kruskal-Wallis test.   PAPS: primary antiphospholipid syndrome; SAPS: secondary antiphospholipid syndrome; Hc: healthy control. Th1:  T helper 1 cells; Th2: T helper 2 cells; Th17: T helper 17 cells; Treg: regulatory T cells; Th1/Th2: T helper 1 cell/ T helper 2 cell ratio; Th17/Treg: T helper 17 cell/regulatory T cell ratio. *P<0.05, **P<0.01, ***P<0.001 | | | | | | |

\

| **Table 3:** Cytokine levels (pg/ml) in PAPS group (A) and SAPS group (B) | | | |
| --- | --- | --- | --- |
| Cytokine levels (pg/ml) | PAPS group (A)  (n=7) ^a^ | SAPS group (B)  (n=30) ^b^ | P-value |
| IL-2 | 2.36 (1.28-3.39) | 2.55 (1.95-3.57) | 0.531 |
| IL-4 | 3.00 (1.46-4.48) | 2.86 (1.83-4.68) | 0.835 |
| IL-6 | 5.36 (3.55-11.39) | 6.60 (5.11-12.28) | 0.370 |
| IL-10 | 4.71 (2.86-5.55) | 5.69 (3.94-8.38) | 0.032* |
| IL-17 | 7.88 (4.80-22.74) | 7.50 (1.81-17.15) | 0.624 |
| IFN-γ | 3.39 (2.79-6.94) | 4.22 (2.49-7.11) | 0.865 |
| TNF-α | 3.50 (2.57-6.93) | 3.01 (1.82-5.84) | 0.719 |
| a means that 5 sets of data are lost.  b means that 25 sets of data are lost.  Results are expressed as the median and 25th and 75th percentiles.  Statistics: Mann-Whitney U test.  PAPS: primary antiphospholipid syndrome; SAPS: secondary antiphospholipid syndrome; IL-2: interleukin-2; IL-4: interleukin-4; IL-6: interleukin-6; IL-10: interleukin-10; IL-17: interleukin-17; INF-γ: interferon-γ; TNF-α: tumor necrosis factor-α. *P<0.05, **P<0.01, ***P<0.001 | | | |

| **Table 4:** Correlations between IL-2,4,6,10 and clinical and laboratory characteristics of APS patients | | | | | | | | |
| --- | --- | --- | --- | --- | --- | --- | --- | --- |
|  | IL-2 | | IL-4 | | IL-6 | | IL-10 | |
|  | r | p | r | p | r | p | r | p |
| ESR | -0.085 | 0.623 | 0.162 | 0.345 | 0.469 | 0.004** | 0.339 | 0.043* |
| CRP | -0.089 | 0.622 | 0.205 | 0.252 | 0.670 | ＜0.001*** | 0.258 | 0.147 |
| WBC | 0.198 | 0.241 | 0.080 | 0.639 | -0.068 | 0.691 | 0.112 | 0.511 |
| Hb | 0.098 | 0.564 | -0.137 | 0.419 | -0.330 | 0.046* | -0.199 | 0.239 |
| PLT | 0.026 | 0.877 | 0.002 | 0.992 | 0.530 | 0.001** | 0.058 | 0.735 |
| LY | 0.092 | 0.594 | -0.011 | 0.948 | 0.024 | 0.892 | -0.112 | 0.516 |
| aCL | -0.420 | 0.010* | -0.392 | 0.016* | -0.286 | 0.086 | -0.331 | 0.046* |
| aβ2GPI | -0.288 | 0.084 | -0.231 | 0.170 | -0.178 | 0.292 | -0.217 | 0.196 |
| C3 | 0.104 | 0.577 | 0.073 | 0.695 | 0.095 | 0.610 | 0.149 | 0.422 |
| C4 | 0.119 | 0.522 | -0.073 | 0.698 | -0.083 | 0.656 | 0.156 | 0.403 |
| D-Dimer | -0.112 | 0.522 | 0.167 | 0.338 | 0.218 | 0.208 | 0.289 | 0.092 |
| Fibrinogen | -0.336 | 0.042* | -0.143 | 0.399 | 0.372 | 0.023* | -0.033 | 0.848 |
| APTT | -0.225 | 0.181 | -0.337 | 0.042* | -0.132 | 0.435 | -0.254 | 0.129 |
| PT | 0.085 | 0.617 | 0.134 | 0.428 | 0.175 | 0.300 | 0.273 | 0.120 |
| Correlations were assessed using Spearman’s rank test.  ESR: erythrocyte sedimentation rate; CRP:C-reactive protein; WBC: white blood cell; Hb: hemoglobin; PLT: platelet; LY: lymphocyte; aCL: anticardiolipin antibody; aβ2GPI: anti-β2 glycoprotein-I; C3: complement C3; C4: complement C4; APTT: activated partial thromboplastin time; PT: prothrombin time; IL-2: interleukin-2; IL-4: interleukin-4; IL-6: interleukin-6; IL-10: interleukin-10. *P<0.05, **P<0.01, ***P<0.001 | | | | | | | | |

| **Table 5:** Correlations between IL-17,IFN-γ，TNF-α and clinical and laboratory characteristics of APS patients | | | | | | |
| --- | --- | --- | --- | --- | --- | --- |
|  | IL-17 | | IFN-γ | | TNF-α | |
|  | r | p | r | p | r | p |
| ESR | 0.100 | 0.593 | 0.094 | 0.587 | 0.118 | 0.493 |
| CRP | 0.335 | 0.081 | 0.120 | 0.505 | -0.019 | 0.917 |
| WBC | -0.075 | 0.684 | -0.261 | 0.118 | 0.055 | 0.745 |
| Hb | -0.052 | 0.776 | -0.142 | 0.400 | -0.021 | 0.902 |
| PLT | 0.239 | 0.187 | -0.030 | 0.858 | 0.059 | 0.728 |
| LY | 0.003 | 0.989 | -0.179 | 0.295 | 0.066 | 0.703 |
| aCL | -0.479 | 0.006** | -0.339 | 0.040* | -0.302 | 0.070 |
| aβ2GPI | -0.143 | 0.434 | -0.196 | 0.244 | -0.116 | 0.493 |
| C3 | 0.440 | 0.025* | 0.122 | 0.513 | 0.071 | 0.704 |
| C4 | 0.220 | 0.280 | 0.172 | 0.354 | 0.058 | 0.756 |
| D-Dimer | 0.081 | 0.670 | 0.047 | 0.790 | 0.035 | 0.843 |
| Fibrinogen | 0.380 | 0.032* | 0.021 | 0.901 | -0.077 | 0.649 |
| APTT | -0.236 | 0.193 | -0.120 | 0.480 | -0.251 | 0.133 |
| PT | 0.360 | 0.043* | 0.323 | 0.051 | 0.149 | 0.379 |
| Correlations were assessed using Spearman’s rank test.  ESR: erythrocyte sedimentation rate; CRP:C-reactive protein; WBC: white blood cell; Hb: hemoglobin; PLT: platelet; LY: lymphocyte; aCL: anticardiolipin antibody; aβ2GPI: anti-β2 glycoprotein-I; C3: complement C3; C4: complement C4; APTT: activated partial thromboplastin time; PT: prothrombin time; IL-17: interleukin-17; INF-γ: interferon-γ; TNF-α: tumor necrosis factor-α. *P<0.05, **P<0.01, ***P<0.001 | | | | | | |

| **Table 6:** In the SAPS group, complement C3 levels of different antibody subgroups | | | |
| --- | --- | --- | --- |
| Autoantibodies  Numbers:  (positive: negative) ^a^ | complement C3 levels (g/L) | | P-value |
|  | positive | negative |  |
| ANA (40:6) | 0.61±0.21 | 0.90±0.35 | 0.007** |
| RF (10:36) | 0.62±0.15 | 0.65±0.28 | 0.723 |
| Anti-ENA (24:22) | 0.58±0.21 | 0.72±0.28 | 0.049* |
| Anti-SSA or SSB (23:23) | 0.57±0.21 | 0.72±0.27 | 0.035* |
| Anti-nRNP /Sm (9:37) | 0.58±0.18 | 0.66±0.27 | 0.376 |
| Nucleosome or histone (12:34) | 0.55±0.12 | 0.68±0.28 | 0.125 |
| Anti-dsDNA (10:36) | 0.55±0.12 | 0.67±0.28 | 0.189 |
| ANCA (12:34) | 0.58±0.18 | 0.67±0.27 | 0.319 |
| a means that serum complement C3 data were available for only 46 of 55 patients in the SAPS group.  Results are expressed as the mean ± standard deviation.  Statistics: independent-samples t-test.  ANA: antinuclear antibody; RF: rheumatoid factor; Anti-ENA: anti-extractable nucler antigen; Anti-dsDNA: anti-double-stranded DNA; ANCA: anti-neutrophil cytoplasmic antibody. *P<0.05, **P<0.01, ***P<0.001. | | | |

| **Table 7:** In the SAPS group, complement C4 levels of different antibody subgroups | | | |
| --- | --- | --- | --- |
| Autoantibodies  Numbers:  (positive: negative) ^a^ | complement C4 levels (g/L) | | P-value |
|  | positive | negative |  |
| ANA (40:6) | 0.11 (0.08-0.17) | 0.20 (0.15-0.28) | 0.047* |
| RF (10:36) | 0.11 (0.08-0.19) | 0.12 (0.07-0.19) | 0.865 |
| Anti-ENA (24:22) | 0.11 (0.08-0.17) | 0.13 (0.08-0.20) | 0.766 |
| Anti-SSA or SSB (23:23) | 0.11 (0.07-0.17) | 0.13 (0.09-0.20) | 0.367 |
| Anti-nRNP /Sm (9:37) | 0.11 (0.08-0.14) | 0.13(0.08-0.20) | 0.461 |
| Nucleosome or histone (12:34) | 0.11 (0.07-0.14) | 0.13 (0.08-0.21) | 0.224 |
| Anti-dsDNA (10:36) | 0.11 (0.08-0.13) | 0.14 (0.08-0.21) | 0.230 |
| ANCA (12:34) | 0.11 (0.07-0.16) | 0.13 (0.08-0.19) | 0.430 |
| a means that serum complement C4 data were available for only 46 of 55 patients in the SAPS group.  Results are expressed as the median and 25th and 75th percentiles.  Statistics: Mann-Whitney U test.  ANA: antinuclear antibody; RF: rheumatoid factor; Anti-ENA: anti-extractable nucler antigen; Anti-dsDNA: anti-double-stranded DNA; ANCA: anti-neutrophil cytoplasmic antibody. *P<0.05, **P<0.01, ***P<0.001. | | | |
